# Book Reviews

**Published:** 1893-11

**Authors:** 


### Book Reviews.

BUREAU OF EDUCATION—Circular of Information No. 2, 1892. Benjamin Franklin and the University of Pennsylvania. Edited by FRANCIS NEWTON THORPE, Ph. D., Professor of American Constitutional History in the University of Pennsylvania. Washington Printing Office. 1893.

In a former number of the JOURNAL was noticed the first number of the Bureau of Education—a history of the University of Virginia, founded by Jefferson. We have now before us number two of the Bureau, a volume of 450 pages, giving a complete history of the University of Pennsylvania and an interesting sketch of Franklin, prepared by Professor Francis Newton Thorpe. We know of no more admirable narrative of the philosopher, philanthropist, and friend of education than this review of his life. Familiar as is the world with the career of Franklin as a statesman and scientist, his larger public services have somewhat overshadowed his labors in the cause of education. The volume before us reveals him as the inspiration of the best educational influences and labors in his adopted state. The glory and the pride of Pennsylvania in her University and in her larger charitable institutions are closely associated with the name of her greatest citizen. Franklin was the first president of the board of trustees of the University, and for a half century was an active member. His early suggestion, in 1749, of the need of higher education indicates the scope and breadth of his thought. "The great end and aim of all learning is to serve mankind, one's country, friends, and family." His theory of education, and of life, was intensely utilitarian, and looked rather to social than political results, in contrast to Jefferson, whose basal theory was political rather than social. His proposal for founding an Academy in Philadelphia was responded to both by citizens and the local government.

Out of this beginning grew the Pennsylvania College, and, finally, the Academy, the College, and the Charity School connected with it, were merged in 1791, under a new charter in the University of Pennsylvania, which has become one of the most important educational institutions of the country. A large part of the volume before us is devoted to a history of its several departments. Of these one is a University Hospital—a rare, if not unique, feature in an educational institution, an adjunct of the medical department. The history of this department reads like a fascinating romance. It was begotten in the enthusiasm of three young physicians, who inspired both private and public wealth to so richly endow the Hospital that with a property now of over a millon dollars in value, it is one of the noblest and most beneficent monuments of charity in the world. It is refreshing to read of the several $50,000 legacies, another of $80,000 and one of $100,000, some of them from physicians connected at some time with the department, who have so embalmed their names in grateful memories. One such legacy was that of the venerable Dr. George B. Wood, the first president of the Hospital, who left $60,000 to its permanent endowment fund.

The following statement has its interest: "Upon the young men of the board was to fall the oversight of the administration of the hospital."

The Government of the United States is rendering invaluable service to the interests of education in every department of learning by this series of educational circulars. They promise to give a history of the leading institutions of learning which will stimulate the best thought and action of their teachers, and, we trust, that liberality of private wealth which shall place our hospitals and universities on a basis of independence and usefulness equal to their needs and worthy the American people.

THE ART OF PRESERVING HEALTH. Outlines of Practical Hygiene, adapted to American conditions. By C. GILMAN CURRIER. M. D., Visiting Physician to the New York City Hospitals ; Fellow of the New York Academy of Medicine ; Member of the New York Pathological Society ; Member of the American Medical Association, etc., etc. One large octavo volume, 468 pages, illustrated, $2.75. New York : E. B. Treat, 5 Cooper Union. 1893.

The appearance of medical works on hygiene is one of the healthy signs of the times. Observing doctors believe that works of this character are now in demand, that the requisition is to be an increasing one, and, therefore, the books are to be produced.

The book in question gives evidence that its author felt the pressure of the demand, and we wish we might add that the book also shows that its author had something to say about hygiene of such a nature as to justify its appearance, regardless of any temporary conditions of the book market. The fact is, we turn the pages of Dr. Currier's outlines of hygiene with the feeling that here is a case of over-production ; the case of a writer having a superficial acquaintance with his subject, but innocent of enthusiasm, convictions, or knowledge ; right in his feeling that in the matter of the art of preserving health, the people need enlightenment, and amiable in his desire to stand in the gap and show the light.

We suspect that if the author had taken that advantage of the vigorous exercise and the cold baths which he so feebly recommends, and so drearily cautions against, he could and would have produced a more virile and useful book. It is possible the writer is prejudiced against both book and its author from what he says of his relations to the public institutions of his city. The visiting physician to the New York City *hospitals* must of necessity have but a limited portion of his time for the study of hygiene. The use of titles like the above, high-sounding, unmeaning, and misleading, seems un-American, unhygienic, and unprofessional. The inability of the author to grasp his subject is shown in his remarks upon contagious and infectious diseases, pages 401 to 429, in which the terms contagion, infection, contagious, infectious, transmissible, and communicable, are so interchanged, synonymously used, and abused, as to give the reader a tired feeling and yet a clear sense of the mental aridity of the author.

It is with a profound sense of relief that one turns from the amiable inaninities of Currier to the sturdy, virile pan-American Gihon, and, in a single paragraph, gets a touch of what hygiene is when adapted to American conditions. We would advise those would-be authors, who feel constrained to comply with the demand to write books upon hygiene adapted to American conditions, to read and re-read Gihon's Sanitary Motes and Beams. H. R.

---

The Diseases of the Nervous System. A Text-book for Physicians and Students. By Dr. Ludwig Hirt, Professor at the University of Breslau. Translated, with permission of the author, by August Hoch, M. D., assisted by Frank R. Smith, A. M., (Cantab..) M. D., Assistant Physician to the Johns Hopkins Hospital. With an introduction by William Osler, M. D., F. R. C. P., Professor of Medicine in the Johns Hopkins University, etc. With 178 illustrations. Large octavo, pp. xv.—683. New York: D. Appleton & Co. 1893.

Professor Hirt has been long recognized as one of the ablest writers on neurology in Germany, and the translation of this work will do much toward creating a proper appreciation of his ability by American readers. The year 1893 has been fertile in the production of treatises on nervous diseases, and among the best must be considered the present volume.

The author divides the subject into four divisions, treating respectively of the Diseases of the Brain and its Meninges, including those of the Cranial Nerves; Diseases of the Spinal Cord; Diseases of the General Nervous System; and Diseases of the General Nervous System with Known Anatomical Basis.

The author is very methodic in the treatment of his various subjects and divisions, and herein lies one of the strong points of the book. Instead of beginning with the physiology or histology of the cortex, he commences peripherad, and discusses, first, the diseases of the inner surface of the dura mater, then those of the soft membranes of the brain, following with the various affections of the cranial nerves, beginning with the olfactory and terminating with the hypoglossal. Under the disorders of the vagus nerve are discussed gastralgia, nervous dyspepsia, and esophagismus. The author gives preference to arsenic in the treatment of these disorders.

Basedow's disease, or exophthalmic goitre, is also considered among the disorders of the vagus, contrary to the views of the majority of observers. No tangible reasons are given for this, neither does he state clearly his objections to it being considered a disease of the sympathetic. His mode of treatment is similar to that of most neurologists—namely, systematic galvanic treatment, the cathode being applied to the lower angle of the jaw, over the vagus and sympathetic nerves, while the anode is held over the lower cervical vertebræ. This certainly has given the most brilliant results in this puzzling affection. Whether the vagus is thus benefited or the sympathetic, the author will not state. The chapters on the brain are profusely illustrated, showing the anatomy and the relative positions of the exterior and interior of the brain, with remarks on the physiology and histology of the various parts.

Regarding the use of electricity in these affections, the author states that it must be absolutely admitted that it is possible to act upon the brain with the galvanic current. This view is in contradiction to the ideas of some who claim that the electricity will pass through tissues offering the least resistance, as the scalp.

Regarding the treatment of hemiplegia after cerebral hemorrhage, the author deprecates the administration of internal medicines, and advises the long continued use of galvanism. These cases, he says, "which have been benefited, are numerous enough and they would undoubtedly be met with more frequently if a fair trial were given it more often than is unfortunately the case." It is quite the fashion among general practitioners to disparage these unfortunates from using systematic electricity, or else, perhaps, to advise them to buy the cheap faradic "music-boxes" and begin home treatment.

The chapter on Brain Tumor is nicely written, and full of good common sense, which feature, by the way, crops out in nearly every chapter of the book. For instance, he says that the existence of local symptoms does not always facilitate the diagnosis as much as one might suppose, because they may be due, indirectly, to the general symptoms, is a point well worth remembering ; and, no doubt, some cases of brain tumor, with clearly definable anteoperation lesions, have disgusted both surgeon and neurologist by their absence in the exact position. The author does not take kindly to the affections known as anemia and hyperemia of the cord, regarding the special symptoms of each as altogether too hypothetical. The chapters on Chorea are well written, and deserve careful reading. As to the relation between chorea and rheumatism, the writer believes that there is some obscure association. He says :

> We have to deal with a common noxious agent, an infection which, if chiefly localized in the brain, gives rise to choreic movements, while, if it affects the joints, it causes acute rheumatism in them, and, if affecting the heart, produces endocarditis and myocarditis.

He also states that the possibility that chorea has some connection with epilepsy cannot, *a priori*, be thrown aside. Then epilepsy must be considered an infectious disease, a premise which will hardly be accepted by many neurologists. In the treatment of chorea, the author prefers Fowler's solution of arsenic, generally obtaining a beneficial result in from fifty to sixty days.

Multiple sclerosis, locomotor ataxia, dementia paralytica, syphilis of the nervous system, and the toxic paralyses are discussed under Diseases of the General Nervous System. The chapters are interesting to read, and contain some old information in very attractive language.

On the whole, it is worthy of careful perusal from preface to index, and coupled with well-printed, good paper, reflects credit on the author and on the Appletons. W. C. K.

---

A Manual of Bacteriology. By George M. Sternberg, M. D., Deputy Surgeon-General, U. S. Army; Director of the Hoagland Laboratory (Brooklyn, L. I.); Honorary Member of the Epidemiological Society of London, of the Royal Academy of Medicine of Rome, of the Academy of Medicine of Rio de Janeiro, of the American Academy of Medicine, etc. Illustrated by heliotype and chromo-lithographic plates and 268 engravings. New York: William Wood & Company. 1892.

Americans can point with pride to the work of Sternberg in the field of experimental bacteriology, and especially can they feel proud that the Surgeon-General of the United States Army is the author of such an excellent book as this one. It fairly outrivals anything attempted in the English language, and is exceled by no work claiming to be a treatise in any language.

The author divides the contents into four parts. The first part deals with the Classification, Morphology, and General Bacteriological Technology. After reviewing briefly the history of bacteriology from the time of Leeuwenhoeck to the present, the author mentions the various attempts at classification, adapting that of Baumgarten with slight modifications. The chapters following treat of Staining Methods, the Culture Media, the Various Sterilizing Ovens, the Action of the Germs in Liquid and Solid Media, the Cultivation of Anerobic Bacteria, Experiments upon Animals, and the Various Steps in Photographing Bacteria. The author has had considerable experience in photomicrography, and his advice in regard to the methods employed is very valuable. Perhaps the only fault to find in this chapter is that he advises the foreign instruments, especially *zuiss.* American lenses, equal if not superior to the foreign make, can be obtained at less cost and with the certainty that they are as represented. The photo-micrographs of bacteria following the last chapter are superb.

Part second treats of the General Biological Characters, including an account of the Action of Antiseptics and Germicides. The substances eminently antiseptic and the proportions in which they are efficient are: mercurii iodide, 1 to 40,000; silver iodide, 1 to 33,000; hydrogen peroxide, 1 to 20,000; mercuric chloride, 1 to 14,300; silver nitrate, 1 to 12,500.

In part third is discussed the Pathogenic Bacteria. In regard to the Klebs-Löffler bacillus of diphtheria, the examination of cover-glass specimens and cultures should be made in every suspected case. Among the most favorable media for carrying these bacilli from an infected source to the throats of previously healthy children is mentioned—milks. Hence, the great caution which should be observed by the health authorities regarding the milk supply of cities.

Part fourth is devoted to the Non-Pathogenic Bacteria. In all, some 489 species of bacteria are described in this work, of which 158 species are considered as pathogenic, and 331 non-pathogenic. The bibliography is very complete; 2,582 references to the literature on this subject are given. A complete index adds much to the value of the book.

In a work like this, no review can do ample justice; a careful perusal of its pages is necessary to form any judgment of its worth and excellence. Every physician who tries to keep abreast of the progress of medicine should have Sternberg's Bacteriology in his library, and make frequent consultations with it.

The author is fortunate in having William Wood & Company for his publishers, for no cleaner work of typography has been seen in many days. The plates are beautiful, and the illustrations are exceedingly well executed. W. C. K.

---

EVIDENCES OF THE COMMUNICABILITY OF CONSUMPTION. By G. A. HERON, M. D. (Glas.), Fellow of the Royal College of Physicians of London; Physician to the City of London Hospital for Diseases of the Chest. London: Longmans, Green & Co., and New York, 15 E. Sixteenth street. 1890.

The great work of the medical profession, just now, is to acquire the conviction that tuberculosis is an infectious disease, and then the greater work of communicating this conviction to the public may begin.

We are glad to welcome this book, and to urge that it be read by every doctor in the land. It gives, in a simple, direct, and pleasing style, what its well-chosen title leads one to expect—Evidences of the Communicability of Consumption. The author is fortunate in his conception of the needs of the profession, and happy in his method of meeting that need by philosophical, logical argument, of which we need a little, and by an array of clinical facts and observations, of which we must have a vast amount. Theoretically, the findings and demonstrations of our laboratories should have produced the instant conviction as to the infectiousness of tuberculosis, and we should have been controlled and guided by this conviction for the last ten years; but medicine is an art and never will be entirely scientific, and the rank and file of our busy practitioners—the doctors at this moment in charge of our millions of cases of tuberculosis—will come to regard and treat these cases as producers of a deadly infection, not from the proclamations from headquarters—the laboratories—but from observations and reports from the field—the bedside. It is the good fortune of our author to see this, and to make a valuable addition to this much-needed array of clinical facts.

Particularly practical are Heron's remarks upon the wide prevalence of tuberculosis among the domestic birds, fowls, and animals, whose eggs, milk, or flesh we use for food. We deem it to be the highest duty of those who can, to call and to focus professional attention to the enormous quantity of tuberculous food we are consuming year by year. The public may be trusted to deal with the situation wisely, if the public ever gets the facts.

The serious, earnest spirit of our author is shown in the following from his chapter on prophylaxis:

> It has been said that if the evidence stated in the preceding pages is true, at least two very grave conclusions are forced upon us, because they are its logical consequences. One of these very grave conclusions is, that every man, woman, and child who is tubercular should at once be removed from all possibility of contact, direct and indirect, with the healthy community. The other very grave conclusion is, that all tubercular beasts should be slaughtered, and their carcasses destroyed. I believe these are both logical consequences of the evidence.

The shock we will get from plain speech like this will show us how far we are from the conviction of the infectiousness of tuberculosis.

H. R.

A MANUAL OF ORGANIC MATERIA MEDICA; being a Guide to Materia Medica of the Vegetable and Animal Kingdoms. For the use of Students, Druggists, Pharmacists, and Physicians. By JOHN M. MAISCH, Ph. D., Professor of Materia Medica and Botany in the Philadelphia College of Pharmacy. New (fifth) edition, thoroughly revised. In one very handsome 12mo volume of 544 pages, with 270 engravings. Cloth, $3.00. Philadelphia: Lea Brothers & Co. 1892.

This work has already received favor at the hands of the profession, as indicated by the fact that it has passed to its fifth edition. In its present form, it differs from that of the preceding edition mainly in the fact that the recent observations and investigations on the various articles of materia medica, so far as they come within the scope of this work, have been incorporated, and that the pronunciation of the systematic names of plants and animals has been indicated by marks of accent. The text, also, has been carefully revised, with the view of rendering the characterization of the directions and all other constituents even more precise and valuable for critical research. A number of the new illustrations, too, have been prepared for an elucidation of structural descriptions, and the pharmacopeial directions have been more conspicuously distinguished by the smaller cut, and smaller type for those articles which are not recognized by the Pharmacopeia, or which, at present, are scarcely ever met with in commerce.

This famous teacher states the foregoing, in substance, in his preface to this edition, and we need only call attention to these facts to bring the book sufficiently to the notice of the profession. Sadly enough, the distinguished author has only lately passed from earth, after having rounded up a life of great usefulness in the pursuit of his special field of observation and teaching.

---

TRANSACTIONS OF THE MEDICAL SOCIETY OF THE STATE OF NEW YORK FOR THE YEAR 1893. Edited by FREDERIC C. CURTIS, M. D., Secretary. Standard octavo, pp. 540. Published by the Society. Philadelphia: William J. Dornan, Printer. 1893.

This handsome volume appears with its customary promptness, and is replete with scientific papers and discussions. The first sixty-six pages of the book are taken up with preliminary matter pertaining to the organization of the Society and the minutes of the meeting. We desire to call particular attention to the careful manner in which these minutes are prepared. We are not aware that any State medical society publishes so accurately, in every detail, all the business that is transacted at its meetings.

President Pilcher's address is a scholarly paper that deserves careful reading by every member of the society, and especially by the president and ex-presidents of the American Medical Association.

This volume contains a few useful illustrations, but not as many as we should like to see in a work of this character. We think it would be of decided advantage to authors if they would make greater efforts to illustrate their papers for society transactions. The fact is, these works become text-books, and should be so regarded by every man who publishes a paper in them.

We especially commend the action of the society in formulating elaborate exhaustive discussions on important subjects, and we hope to see this fashion more and more followed as the years advance.

---

Annual of the Universal Medical Sciences. A Yearly Report of the Progress of the General Sanitary Sciences throughout the World. Edited by Charles E. Sajous, M. D., and seventy associate editors, assisted by over two hundred corresponding editors, collaborators, and correspondents. Illustrated with chromo-lithographs, engravings, and maps. Five volumes. The F. A. Davis Company, Publishers, Philadelphia, New York, Chicago, and London. Australian Agency: Melbourne, Victoria. 1893.

The sixth issue of this great annual comes to hand somewhat later than usual. This, however, is not remarkable when we consider the immensity of the work and the great labor that is required to put it out. The delay is probably due to the fact that the editor has temporarily removed to Paris and the necessary complications which would grow out of such a radical change of residence. This, however, will be more than compensated for, we presume, by the fact that Dr. Sajous will be brought into closer touch with the European publications and authors who are drawn upon for contributions and editorial work.

The Annual appears this year without essential change in any of its departments, which we think is wise. The method of publication has already become familiar to the American profession, and the purposes of the work are well served. There is no undertaking in all the literature of medicine that compares with it, and we consider that the price of the volumes is fixed at an astonishingly low rate, considering the vast deal of time and money requisite to prepare them for distribution. We hope the enterprise will be well sustained by the profession of medicine throughout the world.

PSYCHOPATHIA SEXUALIS, with Especial Reference to Contrary Sexual Instinct. A Medico-Legal Study. By DR. R. VON KRAFFT-EBING, Professor of Psychiatry and Neurology, University of Vienna. Authorized translation of the seventh, enlarged and revised, German edition. By Charles Gilbert Chaddock, M. D., Professor of Nervous and Mental Diseases, Marion-Sims College of Medicine, St. Louis ; Fellow of the Chicago Academy of Medicine, Corresponding Member of the Detroit Academy of Medicine ; Associate Member of the American Medico-Psychological Association, etc. In one royal octavo volume, 436 pages. Extra cloth, $3.00, net; sheep, $4.00, net. Sold only by subscription. Philadelphia: The F. A. Davis Co., Publishers, 1914 and 1916 Cherry street.

This book, from its size, should be complete. Nothing pertaining to the contrary sexual instinct should be left out in such a work, and we look for something that is new as an excuse for its publication. We look in vain. There is nothing in it that adds to our knowledge. The conditions described in it have been well written up by Hammond, B. Ball, and other writers, on Folie Erotique, which is the name given by Ball, of Paris.

We doubt if the book will do as much good as it is sure to do harm in a moral sense. As far as establishing psychopathic sexualis as a distinct form of insanity, we think the distinguished author has failed in his purpose. We still believe these erotic feelings, and sensations, and delusions, to be, in a large proportion of cases, symptoms of various and well-recognized forms of insanity. J. W. P.

---

TRANSACTIONS OF THE AMERICAN ASSOCIATION OF OBSTETRICIANS AND GYNECOLOGISTS. Volume V., for the year 1892. Edited by WILLIAM WARREN POTTER, M. D., Secretary, and published by the Association. Octavo, pp. 529. Philadelphia : William J. Dornan, Printer. 1893.

This is one of the volumes that it is a pleasure to possess and to notice in the columns of a medical journal. This association has shown itself to be possessed of great vigor and cohesiveness. It has risen above a systematic and well-planned adverse criticism and detraction so completely as to conquer all opposition. There is no more handsome volume of society transactions published anywhere than this association annually puts forth, and the present one is fully up to its high standard of excellence in every particular. It contains a number of excellent illustrations ; but we should be pleased to see this part of the book improved upon very materially. Authors of papers should remember that society transactions are in reality text-books that often go into the hands of general practitioners of medicine ; hence it is greatly to their individual advantage, as well as to the credit of the associations to which they belong, to bring out illustrations of their topics wherever practicable. This book is printed on tinted paper with clear type, and commends itself to the study of every thoughtful gynecologist, obstetrician, and abdominal surgeon.

---

Elements of Human Physiology. By Ernest H. Starling, M. D., Lond., M. R. C. P., Joint-Lecturer on Physiology at Guy's Hospital, London ; Member of the Physiological Society, etc. With 100 illustrations. Small 8vo, pp. 437. Philadelphia : P. Blakiston, Son & Co. 1892.

Here is one of the little volumes that it is a pleasure to examine. Not all condensations of medical literature are such as to command the favor, not to say the praise, of the reviewer ; but when physiology is correctly taught in a condensed and compact form, we have no hesitation in according to the successful author his full meed of praise. The fact is, that students cannot be taught too much physiology, and all should adopt a systematic method of acquiring a knowledge of this subject. That is the real purpose of hand-books and other more elaborate treatises. The illustrations of this volume are carefully drawn, for the most part, although their number might have been increased with propriety. We think there is great value in accurate drawings and photographs for the illustration of an author's meaning. We feel disposed to encourage artistic and truthful illustrations, especially in this department of science. This book will command a large sale by reason of the valuable nature of its contents.

---

Elementary Physiology for Students. By Alfred T. Schofield. M. D., M. R. C. S., Late House Physician to the London Hospital ; Special Lecturer, National Health Society. In one handsome 12mo volume of 385 pages, with 227 engravings and two colored plates. Cloth, $2.00. Philadelphia : Lea Brothers & Co.

In this small manual, so the author states in his preface, is made an attempt to present to the student of physiology the leading facts of the science in a fuller way than is generally found in a work of this size, and a somewhat critical examination of the book leaves us with the impression that the author has accomplished, at any rate in a large measure, what he started out to do. There is very much need for the study of physiology in our public schools, and this would be a good text-book for educators to adopt for that purpose. It is well printed and exceedingly well illustrated. The figures, for the most part, illustrate in a very satisfactory manner the portions of the text to which they apply. We have not seen so much accurate physiological information presented in the same number of pages as we find in this treatise. Without an attempt at an analytical review, which is scarcely necessary at this time, we beg to commend the book to the study and examination of all students of physiology.

---

Notes of the Newer Remedies, their Therapeutic Applications and Modes of Administration. By David Cerna, M. D., Ph. D., Demonstrator of Physiology in the Medical Department of the University of Texas, Galveston; formerly Assistant in Physiology, Demonstrator of, and Lecturer on Experimental Therapeutics in the University of Pennsylvania; Fellow of the College of Physicians of Philadelphia; Corresponding Fellow of the Sociedad Espanola de Hygiene of Madrid; Associate Editor of Sajous' Annual of the Universal Medical Sciences, etc. Duodecimo, pp. viii.—177. Price, $1.25. Philadelphia: W. B. Saunders, 913 Walnut street. 1893.

In this book will be found a record of the newer remedies that have been introduced since the last revision of the pharmacopeia. Each remedy is briefly treated, giving after its name, its synonyms and chemical composition; then its physical properties, solubility, therapeutic applications, and mode of administering, are mentioned in their order. This is not considered a work on therapeutics in any sense, but the author says that one of his objects in preparing the book is to keep brief records of the therapeutic applications of the newer remedies, especially of those whose usefulness has been more or less ascertained by clinical investigation. It is an excellent book of reference, and will be found convenient for educators, students, and practitioners.

---

Transactions of the Kentucky State Medical Society. New series. Volume I. Thirty-seventh annual meeting, held at Louisville, May 4, 5, and 6, 1892. Octavo volume, pp. x.—340. Louisville: Printed by John P. Morton & Company. 1892.

Among the valuable scientific work done by State medical societies, that of Kentucky must take the first rank. We are pleased to see that this ancient and honorable organization has returned to the fashion of publishing an annual volume of transactions. This custom has not obtained in this society since 1877; consequently, most of its valuable work has been lost, or, at least, has not formed a substantial addition to the medical literature of the country, as it should have done.

Last year, under a resolution introduced by Dr. McMurtry, a committee of publication was appointed, of which he is the chairman, and the present book is the product of the committee's first labor. It is handsomely printed and bound, and makes a decided addition to a physician's library. We do not understand how any physician in Kentucky could possibly do without it, and it is useful reading for physicians outside the boundaries of that State.

---

A Chapter on Cholera, for Lay Readers. History, Symptoms, Prevention, and Treatment of the Disease. By Walter Vought, Ph. B., M. D., Medical Director and Physician-in-Charge of the Fire Island Quarantine Station, Port of New York; Fellow of the New York Academy of Medicine, etc. Illustrated with colored plates and wood engravings. In one small 12mo volume, 110 pages. Price, 75 cents net. Philadelphia: The F. A. Davis Co., Publishers, 1914 and 1916 Cherry street.

Hardly had this book issued from the press before its talented author succumbed to an infectious fever, said to have been contracted from a patient. There is an especial sadness connected with the death of so brilliant and promising a young physician as the author of this little brochure.

Cholera is a disease which the masses need information upon, and in this monograph may be found ample information connected with its history, symptoms, prevention, and treatment. We think it would be wise to provide Boards of Health with copies of the work to be distributed in their discretion, and to be especially studied by their subordinates who are not medical men.

---

Medical Pocket Atlas. Obstetrics. Part I. Labor delineated in ninety-eight plates. By O. Schaeffer, M. D., Assistant at the University Frauen-Klinik, in Munich. Translated and published under the supervision of J. Clifton Edgar, M. D., Adjunct-Professor of Obstetrics in the University of the City of New York; Attending Physician to the New York Lying-in Hospital; Assistant Obstetric Surgeon to the New York Maternity and to the Emergency Lying-in Hospitals. Formerly Volunteer Interne in the University Frauen-Klinik, of Munich. New York: L. Hydel, Publisher, 212 E. 50th street. 1893.

The plates in this work are excellent expositions of the mechanism of labor, and serve to refresh the memory in regard to many of its perplexities. This atlas is to be especially recommended to those who cannot afford to purchase larger works, and it is furnished at such a low price, that hardly any physician desiring such an aid, need deny himself thereof. There is ample need for improvement in the study of obstetrics by physicians, especially those remote from the centers, and such accurate plates as these serve to refresh the memory on many doubtful points, with very little expenditure of time or labor.

---

MANUAL OF PRACTICAL MEDICAL AND PHYSIOLOGICAL CHEMISTRY. By CHARLES S. PELLEW, E. M., Demonstrator of Physics and Chemistry in the College of Physicians and Surgeons, (Medical Department of Columbia College,) New York. Honorary Assistant in Chemistry at the School of Mines, Columbia College, etc. With illustrations. Pp. 314. New York: D. Appleton & Company. 1892.

Taken in its entirety we consider the work before us an excellent guide for a laboratory course in medical chemistry. We also consider the arrangement of the subjects as not quite logical. We believe that Part IV. and V. should precede Part I.

On page 215, the author says: "Living animal membranes act in the same way as dead ones, or as parchment paper." To a certain extent the statement is true, but we believe that it is misleading, as the living membrane does not act in every respect like a dead one.

The author has supplied a needed manual and is to be congratulated over his success. The publishers have gotten the book up in excellent manner. The plates are splendid and typography fine.
J. A. M.

---

A MANUAL OF THE PRACTICE OF MEDICINE, prepared especially for Students. By A. A. STEVENS, A. M., M. D., Instructor of Physical Diagnosis in the University of Pennsylvania, and Demonstrator of Pathology in the Woman's Medical College, Philadelphia. Illustrated. Philadelphia: W. B. Sanders, 913 Walnut street. 1893.

The author of this book in his preface quotes Pope as saying, "Half of our knowledge we must snatch, not take." We presume Dr. Stevens has followed this precept in his professional life. The fact is evident at a glance that knowledge or information is fired at us in this book in the most laconic manner. The title-page announces that the work is prepared especially for students, but of what avail is it to students to have such snatches of the practice of medicine taught them as they will glean from this work? The only purpose the book can serve is to prepare students through the brain-cramming process, for final examination. A knowledge obtained in this way is generally of a very poor order, and while we have nothing but kindness for the author, we still think he has made a mistake in publishing such a work on such a subject.

THE CHRONIC DISORDERS OF THE DIGESTIVE TUBE. By W. W. VAN VALZAH, A. M., M. D., formerly Demonstrator of Clinical Medicine, Jefferson Medical College. Octavo volume, pp. iv.—151. New York: J. H. Vail & Co. 1893.

This monograph has, for the most part, appeared in some of the medical journals within the past year. The author has grouped his contributions in a single volume, which makes them more readily accessible to those who desire to pursue the study of this subject and to possess all valuable literature thereupon.

Diseases of the digestive tract are constantly increasing, and command more attention than formerly; hence, all literature of excellent quality is hailed with delight. But we cannot commend this book as containing anything new or that has not been said before, perhaps, quite as well.

---

MATERIA MEDICA AND THERAPEUTICS. By L. F. WARNER, M. D., Attending Physician St. Bartholomew's Dispensary, New York. Being Vol. V. of the Students' Quiz Series. Pocket size, 244 pages, $1.00. Philadelphia: Lea Brothers & Co. 1892.

The author of this compend announces that it is based upon the works of such acknowledged authorities as Brunton, Bartholow, Wood, Bruce, Edes, and Biddle, and upon the compiler's own notes of the didactic lectures of Professor Peabody, of New York.

Materia medica has to be obtained largely by memorizing, and sometimes a little work of this kind becomes useful in that direction. It furnishes some excuse, at all events, for the publication of a quiz-book like the one before us, which is one of the best of its kind.

